# A Brief Overview of Neurosurgical Management for Breast Cancer Metastasis

**Published:** 2020-05-11

**Authors:** Brandon Lucke-Wold, K Scott

**Affiliations:** Department of Neurosurgery, University of Florida, USA

**Keywords:** Stage IV breast cancer, Brain metastasis, Neurosurgery, Stereotactic radiation, Immunotherapy

## Abstract

Despite advances in chemotherapy and radiation, stage IV breast cancer presents a serious challenge to clinicians in light of the continued poor outcomes for patients. Stage IV breast cancer frequently metastasizes to the brain often necessitating neurosurgical intervention. The goals of the neurosurgeon are to adequately address metastatic disease to the central nervous system, limit morbidity for the patients, while preserving as much neurologic function as possible, and to help guide next steps regarding need for radiation and immunotherapy. In this review, we provide a background overview of the role of neurosurgery in managing stage IV metastatic breast cancer involving the brain, discuss what is known about brain metastasis, and highlight avenues for future study and investigation.

## Background

**Figure 1:**
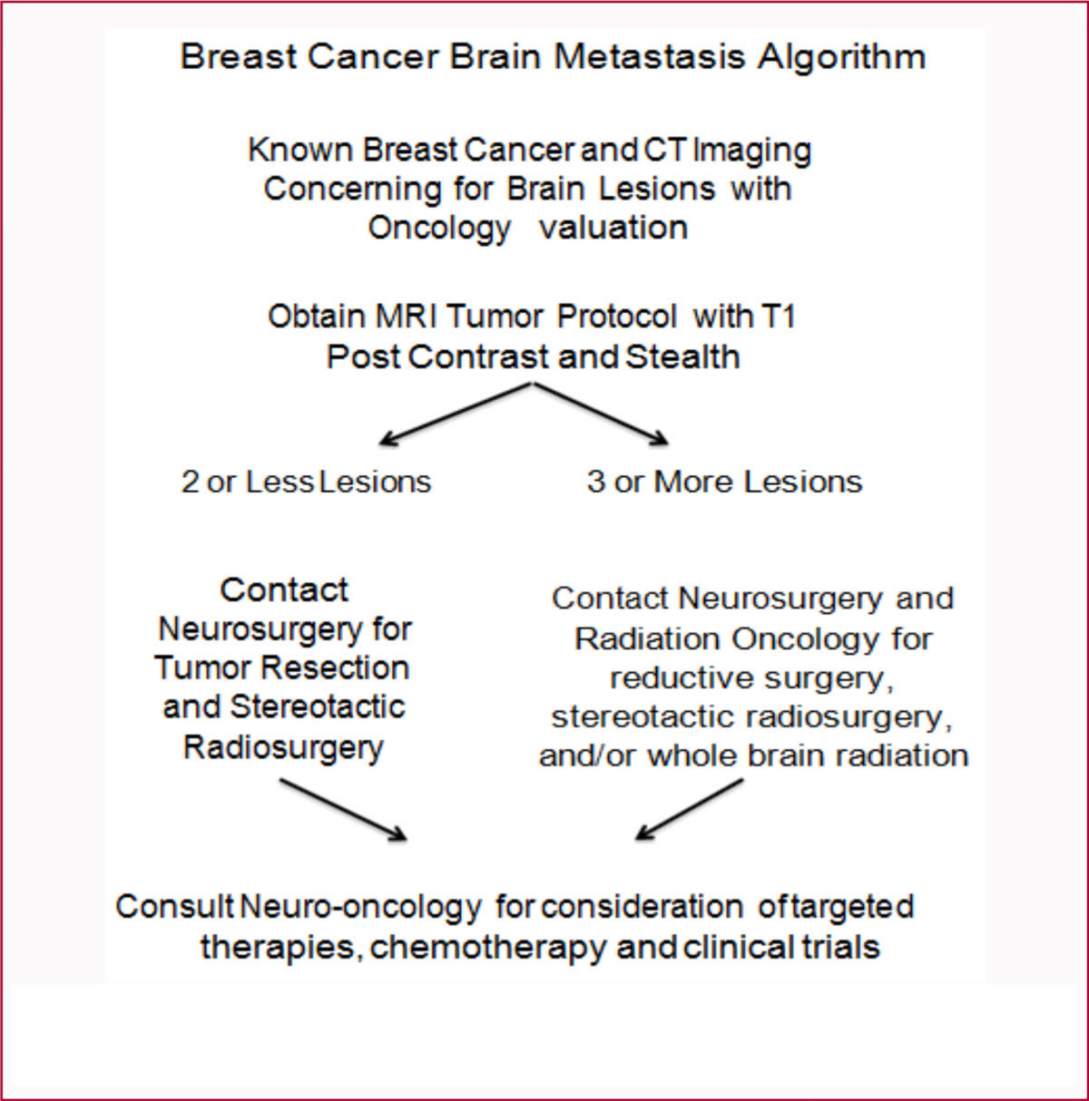
Algorithm for neurosurgical management of breast cancer metastasis.

Among patients with breast cancer about 10–15% will develop metastases [1]. Patients can present with headaches, seizures, cranial nerve deficits, and confusion, depending on tumor location, thus warranting neurosurgical evaluation. In spite of vast improvements in therapeutic options for the treatment of breast cancer to brain metastasis that have taken place between 1985 to 2019, breast cancer metastasis is still associated with poor overall survival, with reports of a 1-year survival rate of 20% [2,3]. Although guidelines vary, historically single lesions or two easily accessible lesions were targeted for gross total surgical resection. More recently as medicine has continued to advance, some groups have expanded resection to as many as 5 lesions. The ultimate decision is based on tumor variables and clinical picture for the patient. The patients with resected tumors have increased survival when stereotactic radiosurgery is provided to the tumor bed following resection [4]. The currently accepted treatment of multi lesion metastasis (>3 brain metastases) is reductive surgery where indicated for symptomatic lesions, radiosurgery targeting as many lesions as deemed safe, or whole brain radiation for large or innumerable lesions in order to reduce cerebral edema [5] (Figure 1). Co- management by neuro-oncology for initiation of chemotherapy and clinical trials is indicated for these patients.

Improvements in personalized medicine have provided additional options for some types of breast cancer. Targeted therapies with trastuzumab and lapatinib for Her2- positive patients have improved survival outcomes [6]. Patients receiving radiosurgery combined with lapatinib had increased average survival to 27.3 months [7]. Case reports have also shown that EGFR positive breast cancer might be responsive to lapatinib and capecitabine [8]. Ongoing investigations are being conducted for trastuzumab and new immunotherapy options with checkpoint inhibitors [9,10].

## Metastasis to the Brain

Despite vast advances in our understanding of the biomechanics and the pathophysiology behind breast cancer metastasis, the exact mechanism of spread still remains a topic of ongoing debate. While cancer metastasis is a highly complex process brought about through spontaneous genetic adaptations developed by rapidly dividing tumor cells, there does appear to be established principles upon which this process functions as will be highlighted below Semanza pioneered a five-stage system for the mechanics of blood or lymphatic vessel metastasis. It consists of intravasation, circulation, margination, extravasation, and colonization [11]. The process involves a cascade of signals within the tumor cell microenvironment that ultimately leads to tumor angiogenesis, cell migration, and eventually extravasation through the vascular wall into the target organ. It is estimated that the hypoxic tumor environment stimulates the production of Hypoxia Inducible Factor-1 (HIF-1) triggering a cascade of pro- angiogenic cytokines such as Tumor Necrosis Factor alpha (TNFα), Vascular Endothelial Growth Factor (VEGF) and Interleukin-8 (IL-8) [12]. After angiogenesis occurs, metastatic cell migration is promoted through numerous signaling pathways, such as the upregulation of chemokine receptors CXCR4 and CCR7. These pathways play a role in determining preferential metastatic destination to end target tissues [13]. Cell migration to the target organ is stimulated *via* an Epithelial-to-Mesenchymal Transition (EMT), the actual mechanisms of which are poorly understood. What is known is that the process involves alteration of the adherens junction *via* down regulation of epithelial cell markers such as E-cadherin, and the upregulation mesenchymal markers such as N-cadherins, thus allowing cell separation and reduced cell-cell adhesion [14]. Once a metastatic cell reaches the circulation, the cell binds to coagulation factors and circulates as embolic material. This embolic particle embeds into a capillary wall and subsequent invasion ensues [15]. The subtype of breast cancer that is involved further specifies metastatic site predisposition. These subtypes are classified as luminal A, luminal B, human epithelial growth receptor type 2 (HER-2), basal-like, and claudin-low [16].

The site specificity of cancer metastasis being dictated by the tumor cell compatibility with the end target tissue is a common notion, and was first suggested in Paget’s “seed and soil” hypothesis [17]. We see this trend in breast cancer metastasis, *via* studies examining the predisposition of breast cancer type and metastatic behavior. In fact, some studies have shown that breast cancer metastasis to the brain is increased in triple-negative and in HER-2+ subtypes in addition to cells exhibiting high expression of nestin, promonin-1, CK-5 and WNT/*β*-catenin signaling [18]. Others have shown factors such as younger age and higher tumor grade to correlate with brain metastasis [19]. One of the biggest questions regarding brain metastasis is the mechanism behind how Circulating Tumor Cells (CTC) penetrates the blood-brain-barrier to deposit in brain capillaries. From gene expression and functional analysis studies, cyclooxygenase COX_2_, Epidermal Growth Factor Receptor (EGFR) ligand HBEGF, and alpha-2,6-sialyltransferase ST6GALNAC5 have all been identified as mediators of breast cancer cell extravasation into the brain [20]. Further work is needed on how these factors contribute to blood brain barrier breakdown.

Overall, the current body of evidence demonstrates an immensely complex and dynamic cascade involving countless transcriptional modifications and signaling proteins that allow breast cancer to metastasize to the brain. These molecular pathways permit both the predictability and spontaneity associated with cell metastasis and warrant further investigation.

## Systemic Treatment Options

Systemic treatment is commonly implemented in conjunction with surgical resection or radiotherapy in the management of brain metastases of breast cancer and helps to reduce recurrence rates. Among ER positive patients’ hormonal therapy (tamoxifen, megestrol acetate, and aromatase inhibitors) can be offered [21]. Many cytotoxic chemotherapy agents (cyclophosphamide, fluorouracil, methotrexate, doxorubicin, etoposide, capecitabine, etc.) can also be implemented likely due to the increase permeability of the Blood-Brain-Barrier (BBB) [22]. Targeted therapies such as, Lapatinib (EGFR/ Her2), Trastuzumab (Her2), Neratinib (Her2), and Bevacizumab (VEGF) have each shown efficacy in halting crucial mechanistic steps in the pathogenesis of breast cancer metastases outlined earlier [23,24].

## Radiotherapy Management Options

Current recommendations for the management of patients with brain metastasis of breast cancer vary based on the receptors expressed (ER, PR, HER2) and the degree of metastasis. Per ASCO guidelines, patients with a single central nervous system lesion that is surgically accessible, surgery should be offered with Stereotactic Radiosurgery (SRS) in post-op, whereas, SRS alone should be offered for patients who are unfavorable surgical candidates. If SRS is unavailable, Whole-Brain Radiotherapy (WBRT) or Fractionated Stereotactic Radiotherapy (FSRT) should be offered to reduce the recurrence risk [25]. Furthermore, serial imaging should be conducted every 2 to 4 months to monitor for brain failure. Patients with two to four metastases should receive resection for larger accessible lesions plus postoperative radiotherapy and SRS for smaller lesions. WBRT, FSRT, and SRS are also reasonable approaches for lesions larger than 3 cm. SRS remains controversial for patients with 5 to 10 sites of brain metastasis however is still a reasonable alternative to WBRT [26]. For patients with very diffuse metastasis and a favorable prognosis WBRT should be offered; while WBRT and palliative care can be offered for those with a poor prognosis.

## Innovation: The Way Forward

As treatment modalities continue to evolve, one promising approach is Laser Interstitial Thermal Therapy (LITT). The benefit of LITT is that local control of tumor burden can be obtained in a quicker manner than radiation treatment [27]. Another emerging field is immunotherapy. Many groups are looking at how breast cancer metastasis has shared molecular characteristics despite subtypes. These shared pathways thus offer novel avenues for immunotherapy targets (many that are currently being investigated) [28]. Some innovative thinkers have even proposed stem cells to tackle the challenge of brain metastasis from breast cancer [29]. A few groups have even tried prophylatic whole brain radiation in advanced breast cancer with mixed results [30].

Another up and coming therapy option is the use of Focus Ultrasound (FUS) on brain cancer metastases. FUS had demonstrated immense versatility with therapeutic utility in its ability for thermal ablation, penetration of the BBB, immunomodulation, sonoporation, sensitization to chemotherapy, and many others that currently remain under study [31]. Furthermore, studies in animal models have already begun to show that FUS with microtubules has the potential to disrupt the BBB and aid in the delivery of targeted antibody therapies, such as trastuzumab, producing better responses in models of breast cancer metastases [32]. These exciting new options remain in early clinical trials but demonstrate up and coming options for patients in the future.

## Summary

The adopted standard for many centers for 2 or less brain metastases from breast cancer is surgical resection followed by stereotactic radiosurgery. Novel adjunctive chemotherapy is now being considered at multiple institutions. For 3 or more metastases to the brain, stereotactic radiation or whole brain radiation are the clinical options, with resection of a symptomatic lesion if easily accessible. Novel innovations such as immunotherapy with checkpoint inhibitors and LITT that target molecular pathways highlighted above may prove promising in the future. Further research regarding improved treatment modalities is warranted.
